# Diazepam effects on local cortical neural activity during sleep in mice

**DOI:** 10.1016/j.bcp.2021.114515

**Published:** 2021-09

**Authors:** Laura E. McKillop, Simon P. Fisher, Linus Milinski, Lukas B. Krone, Vladyslav V. Vyazovskiy

**Affiliations:** Department of Physiology, Anatomy and Genetics, University of Oxford/Sleep and Circadian Neuroscience Institute, United Kingdom

**Keywords:** Diazepam, Benzodiazepines, Sleep, Electroencephalogram, Local field potentials, Multiunit activity

## Abstract

GABA-ergic neurotransmission plays a key role in sleep regulatory mechanisms and in brain oscillations during sleep. Benzodiazepines such as diazepam are known to induce sedation and promote sleep, however, EEG spectral power in slow frequencies is typically reduced after the administration of benzodiazepines or similar compounds. EEG slow waves arise from a synchronous alternation between periods of cortical network activity (ON) and silence (OFF), and represent a sensitive marker of preceding sleep-wake history. Yet it remains unclear how benzodiazepines act on cortical neural activity during sleep. To address this, we obtained chronic recordings of local field potentials and multiunit activity (MUA) from deep cortical layers of the primary motor cortex in freely behaving mice after diazepam injection. We found that the amplitude of individual LFP slow waves was significantly reduced after diazepam injection and was accompanied by a lower incidence and duration of the corresponding neuronal OFF periods. Further investigation suggested that this is due to a disruption in the synchronisation of cortical neurons. Our data suggest that the state of global sleep and local cortical synchrony can be dissociated, and that the brain state induced by benzodiazepines is qualitatively different from spontaneous physiological sleep.

## Graphical abstract

**Figure:**
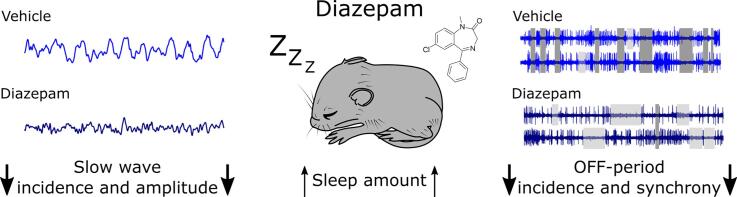

## 1 Introduction

Benzodiazepines are frequently prescribed as sedatives, anxiolytics and anticonvulsants, with 5.2% US adults aged 18–80 reported to be using benzodiazepines in 2008 [53]. Benzodiazepines are commonly used for the management of epileptic seizures including status epilepticus [49], [65], and anxiety disorders such as panic disorder and generalised anxiety disorder [4], [39], [50]. Benzodiazepines also have notable sedative and sleep-inducing effects, which have led to them being used as hypnotics for the treatment of insomnia [9], [30], [70]. However, it was noted that benzodiazepines can enhance the impairments in daytime functioning commonly reported in individuals experiencing sleep disturbances, affecting performance, cognition and memory, as well as increasing the risk of accidents, particularly with long-term use [5], [27], [30], [66], [77]. Benzodiazepines are also highly addictive, and patients taking them on a regular basis develop tolerance and prominent withdrawal symptoms, which can ultimately lead to their misuse [48], while chronic use is often ineffective or can lead to impairments in cognition [5], [30], [62]. For these reasons, benzodiazepines and other hypnotic drugs are not generally recommended for the treatment of insomnia and should be limited to short-term use [61], [63], [64], [68]. Despite these risks, benzodiazepines remain a prevalent treatment for sleep disruptions, with pharmacological interventions resulting in similar short-term treatment outcomes for persistent insomnia as compared to behavioural interventions [74], and even low doses reported to produce significant hypnotic effects [72].

Many of the pharmacological interventions for the treatment of sleep disorders including insomnia, act by enhancing gamma-amino butyric acid (GABA) transmission. Multiple populations of GABAergic neurons have been identified as having important roles in the induction and maintenance of sleep throughout the brain, and have been extensively reviewed elsewhere [37]. Briefly these include non-rapid eye movement (NREM) sleep inducing populations in the ventrolateral preoptic area of the hypothalamus, the parafacial zone in the brainstem, the nucleus accumbens and the cortex, and populations associated with rapid eye movement (REM) sleep in ventral medullary reticular formation, lateral hypothalamic area and ventrolateral periaqueductal gray. Of particular relevance to this study, benzodiazepines potentiate inhibitory GABAergic neurotransmission by increasing the affinity for the inhibitory neurotransmitter gamma-amino butyric acid (GABA) to bind to GABA type A receptors (GABA_A_), including containing alpha1, alpha2 and gamma2 subunits which are distributed throughout the brain [29], [28], [78], [79], [80]. It is thought that benzodiazepines act mainly by potentiating GABAergic transmission in subcortical structures controlling the thalamo-cortical networks responsible for the generation of delta oscillations during slow wave sleep, rather than having direct effects [37].

Benzodiazepines, such as diazepam, reduce overall network excitability and lead to characteristic alterations in rhythmic activity. In fact there is recent evidence that diazepam administration in mice induces a slowing of global electroencephalogram (EEG) oscillations in a frequency- and behavioural-state dependent manner [69]. Studies in both humans and animals have shown that benzodiazepineadministration shortens the latency to sleep, increases sleep continuity, inhibits REM sleep, and at the level of EEG spectra leads to increases in fast frequencies such as sleep spindles and stage 2 NREM sleep at the expense of stage 3 slow wave sleep [2], [24], [29], [28], [34], [35], [37], [69], [79], [6], [7], [8]. Evidence in humans has shown that the reduction in sleep slow wave activity (EEG power density between 0.5 and 4 Hz, SWA) persists into the following drug-free night after injection with various benzodiazepines, whereas the effects on the spindle frequency range (10–15 Hz) were recovered by the following day [8]. Interestingly benzodiazepines do not seem to affect the homeostatic regulation of sleep, as evidence suggests they do not greatly affect the time course of SWA, despite reducing absolute EEG power [2]. Theta frequency activity (6–9 Hz) during waking and REM sleep has also been shown to be enhanced in mice injected with diazepam [28], [79]. Diazepam also shifted the theta peak to slower frequencies, especially in the occipital derivation where theta activity is known to predominate due to its proximity to the hippocampus. It is possible that the shift in theta peak may reflect changes in body temperature, as has previously been shown [15], [16]. This is supported by evidence that diazepam decreases body temperature [38].

Thus, current evidence suggests seemingly paradoxical effects of diazepam on sleep, in that it is sleep promoting yet reduces sleep-related global oscillatory EEG activities. An explanation for this could lie in potential local effects of diazepam on neural activity which are not well understood, and are likely to be complex. Electrophysiological characteristics, connectivity patterns, ongoing behaviour and preceding sleep-wake history may all together determine the firing phenotype of specific cortical neurons [3], [20], [31], [41], [51], [56]. It is crucial to further understand the physiological mechanisms underlying the efficacy of these drugs. In this study we investigated the effects of the commonly used hypnotic, diazepam on characteristics of the electroencephalogram, local field potentials and cortical neural activity in laboratory mice. This study aimed to address whether reduced ‘global’ EEG slow-wave activity after diazepam was associated with changes at the local network level.

## 2 Materials and methods

### 2.1 Experimental animals

This study was carried out in n = 8 male C57Bl/6J mice aged 5.23–12.83 months old. Experiments took place on average 26.63 ± 0.78 days post-surgery. Mice were individually housed in custom-made clear plexiglass cages (20.3 × 32 × 35 cm) with free access to a running wheel (Campden Instruments, UK, wheel diameter 14 cm, bars spaced 1.11 cm apart inclusive of bars) and food available ad libitum. Cages were housed in ventilated, sound-attenuated Faraday chambers (Campden Instruments, UK, two cages per chamber). Mice were housed under entrained conditions (standard 12:12 h light–dark cycle, lights on 0900, 120–180 lx). Room temperature and relative humidity were maintained at 22 ± 1  °C and 50 ± 20%, respectively. All procedures conformed to the Animal (Scientific Procedures) Act 1986 and were performed under a UK Home Office Project Licence in accordance with institutional guidelines.

### 2.2 Surgical implantation of recording electrodes

Surgical implantation of recording electrodes was carried out as previously described [14], [20], [42], under aseptic conditions. Briefly, animals were deeply anaesthetised using isoflurane anesthesia (3%–5% induction, 1%–2% maintenance). One day before surgery, animals received dexamethasone (0.2 mg/kg, p.o.). Metacam (1–2 mg/kg, s.c., Boehringer Ingelheim, UK), dexamethasone (0.2 mg/kg, s.c. Aspen Pharmacare, UK), and vetergesic (0.08 mg/kg, s.c, Ceva Animal Health ltd., UK) were also administered preoperatively. Before implantation, EEG screw electrodes were soldered to custom-made head-mount connectors (Pinnacle Technology) and unilaterally implanted into frontal (motor area: anteroposterior 2 mm, mediolateral 2 mm) and occipital (visual area, V1: anteroposterior 3.5–4 mm, mediolateral 2.5 mm) cortical areas. Reference and ground screw electrodes were implanted above the cerebellum and contralaterally to the occipital screw, respectively. Two single-stranded, stainless-steel wires were inserted on either side of the nuchal muscle to record electromyography. All screws and wires were secured to the skull using dental acrylic. Mice were also implanted with a polymide-insulated tungsten microwire array (Tucker-Davis Technologies), consisting of 16 channels (2 rows each of 8 wires), with a wire diameter of 33 μm, electrode spacing 250 μm, row separation L-R 375 μm, a tip angle of 45 degrees and one row of electrodes 250 μm longer than the other to account for the curvature of the brain. Electrodes were implanted into deep layers of the primary motor cortex as this area has been well studied and it is well established that EEG SWA predominates in the frontal cortex [19], [20], [21], [22], [25], [40], [45], [47], [71], [73], [86], [82], [83], [87], [88]. A 1 × 2 mm craniotomy was made contralaterally to the frontal EEG screw (anteroposterior 1.5–2 mm, mediolateral 2 mm) using a high-speed drill and the arrays lowered below the pial surface into deep layers of the motor cortex. A two-component silicon gel (KwikSil; World Precision Instruments) was used to seal the craniotomy and protect the surface of the brain from the dental acrylic used to fix the array to the skull. Animals were all monitored closely after surgery and provided with analgesia as necessary (0.2 mg/kg dexamethasone (Aspen Pharmacare, UK) for 2 days and 1–2 mg/kg metacam (Boehringer Ingelheim, UK) for a minimum of 3 days).

### 2.3 Signal processing

A Tucker-Davis Technologies Multichannel Neurophysiology Recording System was used for data acquisition. Cortical EEG was recorded from frontal and occipital derivations. EEG, EMG, and LFP data were filtered between 0.1 and 100 Hz, amplified (PZ5 NeuroDigitizer preamplifier, Tucker-Davis Technologies), and stored on a local computer at a sampling rate of 256.9 Hz. Extracellular neuronal spike data were recorded from the microwire array at a sampling rate of 25 kHz (filtered between 300 Hz and 5 kHz). OpenEx software (Tucker-Davis Technologies) was used to manually apply amplitude thresholds for online spike detection and to eliminate artifactual waveforms caused by electrical or mechanical noise. Spikes that exceeded this predefined threshold (>2 × noise level, at least − 25 μV) were stored as 46 samples (0.48 ms before, 1.36 ms after the threshold crossing) consisting of both voltage measures and time stamps. LFP, EEG, and EMG data were then resampled offline at a sampling rate of 256 Hz. Custom-written MATLAB scripts (The MathWorks) were used for signal conversion. Data were then transformed into European Data Format using open source Neurotraces software.

**Fig. 1:**
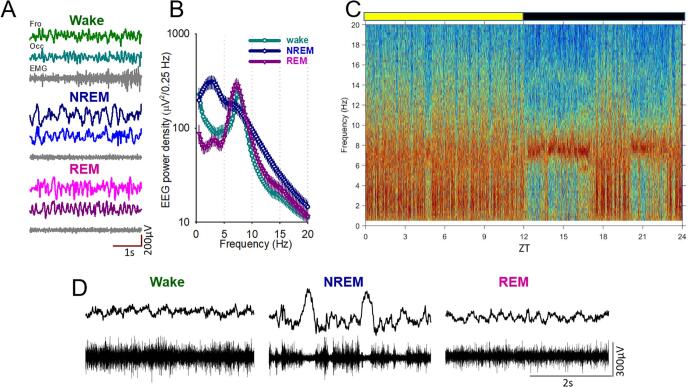
Baseline sleep-wake behaviour and neural activity in mice. A) representative frontal (Fro) and Occipital (Occ) electroencephalogram (EEG), and electromyogram (EMG) signals shown during wakefulness (top), NREM sleep (middle) and REM sleep (bottom). Scale bars represent 1 s and 200 μV. B) Average power spectra between 0 and 20 Hz shown separately for each vigilance state for the occipital EEG signal. n = 8. C) Representative EEG power spectrogram for an individual animal during a 24 h baseline recording. Note: slow frequencies, indicative of NREM sleep mostly occur during the light phase when mice are predominantly sleeping. D) Example LFP and associated multiunit activity during Wake (left), NREM sleep (middle) and REM sleep (right). Scale bars represent 2 s and 300 μV. ZT: Zeitgeber time.

Recordings were subdivided into 4 s epochs and vigilance states scored offline by manual inspection of the signal (SleepSign, Kissei Comtec). Vigilance states were classified as waking (low-voltage, high-frequency EEG with a high level or phasic EMG activity), NREM sleep (presence of EEG slow waves, a signal of a high amplitude and low frequency), or REM sleep (low-voltage, high-frequency EEG with a low level of EMG activity), as shown in the representative traces shown in Fig. 1A. Vigilance state artifacts in at least one EEG or MUA recording channel, were scored as artifacts so that they may be removed from appropriate analyses. After the data were scored, EEG and LFP power spectra were computed by an FFT routine for 4 s epochs (Hanning window), with a 0.25 Hz resolution (SleepSign, Kissei Comtec).

In order to detect slow waves in the LFP, firstly the signal was band pass filtered between 0.5 and 4 Hz (stopband edge frequencies 0.3–8 Hz) with MATLAB filtfilt function exploiting a Chebyshev Type II filter design (The MathWorks Inc, Natick, Massachusetts, USA) [1], [20], [42], [84]. Slow waves were then defined as positive deflections of the filtered LFP signal between two consecutive negative deflections below the zero-crossing, in which the peak amplitude of the wave was larger than the median amplitude detected across all waves. OFF periods were defined as complete cessation of spiking activity across all channels for at least 50 ms. For some of the analyses, only the largest slow waves or longest OFF periods were included (>median + one standard deviation across vehicle and diazepam conditions).

### 2.4 Experimental design

**Fig. 2:**
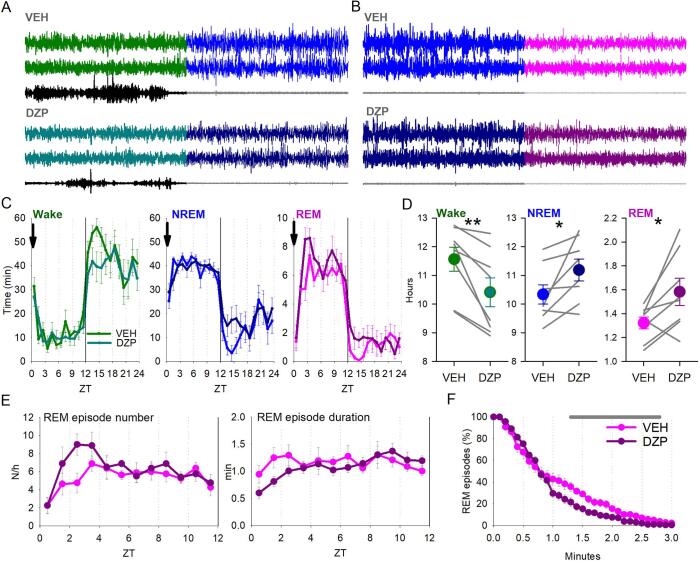
Effects of diazepam injection on vigilance states. A) Example wake to NREM sleep transitions are shown for the vehicle (top) and diazepam (bottom) experimental days. From top to bottom, traces show frontal EEG, occipital EEG and EMG. B) Same as A) but for NREM sleep to REM sleep transitions. C) The time course of wake (green), NREM sleep (blue) and REM sleep (pink) during the 24 h period after either vehicle or diazepam injection at time zero, averaged in 1 h intervals. Arrow indicates the time of injection. n = 8. Repeated measures ANOVA found no interaction and no main effect for injection condition, but only a main effect of time (wake: F(6.6,92.5) = 22.9, p < 0.0001; NREM sleep: F(6.67,93.7) = 19.44, p < 0.0001; REM sleep: F(6.5, 91.1) = 28.67, p < 0.0001). D) Total amount of each vigilance state over the 24 h period of the vehicle and diazepam injections. Data are mean ± SEM, with individual animals also shown as thin lines. n = 8. Repeated measures ANOVA revealed a significant interaction between vigilance state*injection condition (F(1.1,7.9) = 10.57, p = 0.01). Paired t-tests revealed significant differences between vehicle and diazepam injection conditions for each vigilance states (wake: p = 0.007; NREM sleep: p = 0.03; REM sleep: p = 0.03). *p < 0.05, **p < 0.01. E) Number and duration of REM sleep episodes, respectively, over the 12 h light period after injection. Repeated measures ANOVA found no interaction and no main effect for injection condition, but only a main effect of time on the number of REM sleep episodes (F(11,154) = 3.88, p < 0.0001). F) Survival analysis showing the percentage of REM episodes that meet increasing REM sleep duration criteria. Repeated measures ANOVAs with factors time, injection condition and their interaction. Shaded areas above traces indicate p < 0.05. In panels D-G data are mean ± SEM and n = 8. ZT: Zeitgeber time. VEH: vehicle injection. DZP: diazepam injection.

This study was a cross-over design with all animals receiving both an injection of diazepam (3 mg/kg, Hameln Pharmaceuticals ltd, UK) and vehicle (saline with 0.3% Tween 80, Sigma Aldrich (now Merck, Darmstadt, Germany)) in a randomised order, with 96 h between each injection. Diazepam was dissolved in the vehicle and injected at a concentration of 10 mg/ml. Injections were performed at light onset (ZT0). The 24-hours prior to each injection was used as a baseline, while the 24 h after injection was used to assess the effect of injections. Animals were undisturbed throughout the duration of the study and electrophysiological signals were recorded continuously. Example wake to NREM sleep and NREM sleep to REM sleep transitions are shown in Fig. 2A and B, respectively, after both vehicle and diazepam injections.

Data were analysed using MATLAB and SPSS (IBM, released 2016; SPSS Statistics for Windows, version 24.0, IBM). Data are mean ± SEM, unless stated otherwise. Statistical tests used in each analysis are reported in the corresponding figure legend. T-tests and Wilcoxon tests were used where there were only two groups. ANOVAs were used for statistical comparisons of more than two groups. For repeated measures ANOVAs, where sphericity was violated, Greenhouse-Geisser corrected values were instead reported (Welch correction). Bonferroni post hoc tests were used where necessary. For Fig. 5D, data were first log-transformed prior to statistical analysis. In some cases, animals were excluded from specific analyses for technical reasons, as stated in the figure legends. At the end of the study, electrode recording sites were confirmed using previously described histological methodology [20].

## 3 Results

Firstly, we investigated the 24 h baseline period prior to injection. All animals had at least one good EEG channel and EMG that allowed for the identification and scoring of the three vigilance states, as shown in Fig. 1A. In this study, we also looked at the neural multiunit activity associated with local field potentials, which showed classical distinctions between vigilance states. For example, the periodic occurrence of neuronal silence (OFF periods) was typical for NREM sleep, while neurons showed tonic firing during both wake and REM sleep (Fig. 1D). As expected, slow frequencies indicative of NREM sleep occurred predominantly during the light period reflecting the rest period of mice, while animals were mostly awake during the dark, active phase (Fig. 1C). Quantification of average power spectra during the baseline 24 h period across all animals revealed the classic peak in slow wave activity (SWA: 0.5–4 Hz) during NREM sleep and a clear peak in theta range activity during REM sleep (6–9 Hz) (Fig. 1B).

We next investigated the effects of diazepam on overall sleep-wake architecture by comparing the 24 h post diazepam or vehicle injection (Fig. 2). The time course of each vigilance state was not significantly different between injection conditions (Wake: F(1,14) = 3.1, p = 0.18; NREM sleep: F(1,14) = 2.67, p = 0.16; REM sleep: F(1,14) = 4.41, p = 0.24, repeated measures ANOVA, Fig. 2C). However, a repeated measures ANOVA with within subject factors vigilance state and injection condition revealed a significant interaction between the injection conditions (diazepam or vehicle) and the daily time spent in different vigilance states (F(1.1,7.9) = 10.57, p = 0.01; Fig. 2D). Post hoc paired t-tests revealed a significant increase in the total amount of both NREM sleep and REM sleep over the 24 h post injection after diazepam injection as compared to vehicle (NREM sleep: vehicle 10.33 ± 0.34 h, diazepam 11.19 ± 0.34 h, p = 0.03; REM sleep: vehicle 1.32 ± 0.05 h, diazepam 1.58 ± 0.10 h, p = 0.03). The total amount of wake was decreased after diazepam injection (vehicle 11.56 ± 0.42 h, diazepam 10.41 ± 0.45 h; p = 0.007). Though the total amount of REM sleep was increased, no significant difference in the number or duration of REM sleep episodes were identified in the 12 h light period following injection (rest phase, where mice typically sleep) (number: F(1,14) = 0.51, p = 0.49; duration: F(1,9) = 0.54, p = 0.48; Fig. 2E). There was a redistribution of REM episode durations, with fewer longer lasting REM sleep episodes occurring after diazepam injection (Repeated measures ANOVA (Greenhouse-Geisser); Time: F(2.8, 38.1) = 308.68, p < 0.0001; Injection condition: F(1,14) = 3.36, p = 0.09; Interaction: F(2.7, 38.1) = 3.7, p = 0.02; p < 0.05 between 1.3 and 2.4 min; Fig. 2F). In addition, there was no change in the number of NREM sleep episodes after diazepam injection which led to the occurrence of an average of 9.89 ± 0.59 and 11.09 ± 1.02 NREM sleep episodes per hour of NREM sleep for vehicle and diazepam injections, respectively (p = 0.23, paired *t*-test). Notably, the number of brief awakenings per hour of total sleep were not significantly different between vehicle and diazepam injection conditions across the 12 h light period (vehicle: 13.07 ± 1.36 episodes; diazepam: 10.93 ± 1.50 episodes; p = 0.18, paired *t*-test), suggesting that there is no major increase in sleep fragmentation after diazepam injection.

**Fig. 3:**
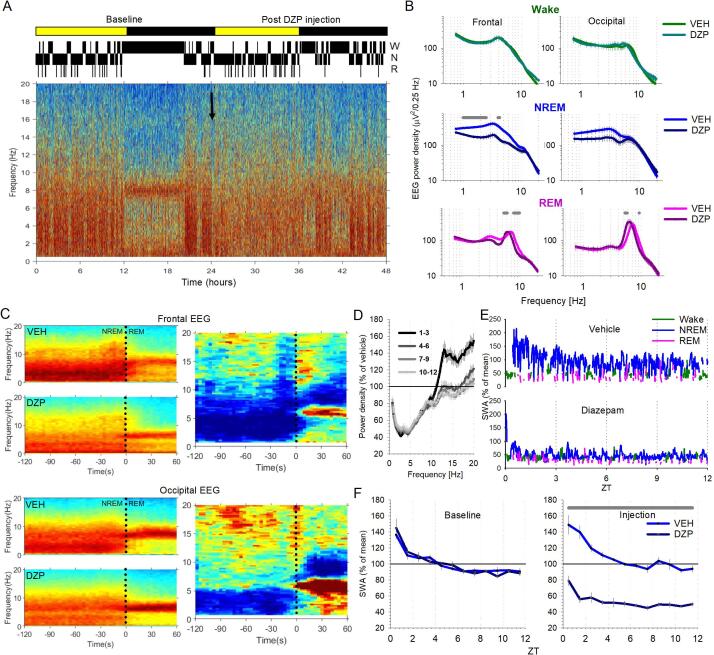
Effects of diazepam on EEG spectra and SWA. A) EEG power spectrogram for a representative animal during the 24 h baseline recording and the 24 h post diazepam injection. Arrow indicates the time of diazepam injection at the start of the light period. Corresponding hypnogram shown above to indicate the vigilance state (top to bottom indicating wake (W), NREM sleep (N) and REM sleep (R), respectively. Note the reduction in slow frequencies post diazepam injection. B) average power spectra for both frontal (left) and occipital (right) EEG derivations are plotted for wake (top, green), NREM sleep (middle, blue) and REM sleep (bottom, pink), separately for vehicle and diazepam injection days. Repeated measures ANOVA with factors frequency, injection condition and their interaction for both the frontal EEG derivation (Wake: freq F(1.4, 14.0) = 24.34, p < 0.0001, injection condition F(1.4, 14.0) = 0.80, p = 0.43, interaction F(1.4, 14.0) = 0.80, p = 0.43; NREM: freq F(1.8, 18.1) = 52.85, p < 0.0001, injection condition F(1,10) = 0.96, p = 0.35, interaction F(1.8, 18.1) = 4.96, p = 0.02; REM: freq F(1.7, 17.2) = 48.84, p < 0.0001, injection condition F(1.7, 17.2) = 48.84, p < 0.0001, interaction F(1.7, 17.2) = 4.98, p = 0.02) and occipital EEG derivation (Wake: freq F(1.4, 16.7) = 23.29, p < 0.0001, injection condition F(1,12) = 0.05, p = 0.84, interaction F(1.4, 16.7) = 0.80, p = 0.42; NREM: freq F(1.6, 19.2) = 41.60, p < 0.0001, injection condition F(1,12) = 0.68, p = 0.43, interaction F(1.6, 19.2) = 3.92, p = 0.05; REM: freq F(1.6, 19.0) = 37.85, p < 0.0001, injection condition F(1,12) = 0.07, p = 0.80, interaction F(1.6, 19.0) = 3.68, p = 0.05). Shaded areas above traces indicate frequency bins where the differences were statistically significant (p < 0.05). Frontal n = 6, occipital n = 7. C) Representative EEG power spectrograms for 120 s before NREM sleep – REM sleep transition and 60 s after transition are shown for the frontal EEG derivation (top) and occipital EEG derivation (bottom) recorded from an individual animal. Dotted line at time 0 corresponds to REM sleep onset. Left panels show the data subdivided into the vehicle (top) and diazepam (bottom) injection days. Right panels show the difference between the vehicle and diazepam conditions. D) Frontal EEG spectral power during NREM sleep after diazepam injection, subdivided into three-hour intervals, expressed as percentage of corresponding NREM EEG spectra after vehicle injection, prior to averaging between animals. Mean values, SEM. n = 6. E) Frontal EEG SWA across the 12-hour light period after vehicle (top) and diazepam (bottom) injection in a representative animal. Data are colour coded according to the vigilance states; NREM sleep (blue), REM sleep (pink) and wake (green). F) The time course of frontal EEG slow wave activity (SWA, 0.5–4 Hz) during NREM sleep across the 12-hour light period of the baseline (left) and injection (right) days. Left panel: baseline SWA expressed relative to the 12 h mean, showing no difference between conditions prior to injection. Right panel: SWA on the injection day. n = 6. Repeated measure ANOVA for baseline days (time F(3.1,31.3) = 23.39, p < 0.0001, injection condition F(1,10) = 0.30, p = 0.60, interaction F(3.1,31.3) = 0.53, p = 0.67) and injection days (time F(3.0,30.2) = 20.9, p < 0.0001, injection condition F(1,10) = 210.26, p < 0.0001, interaction F(3.0,30.2) = 3.71, p = 0.02). Shaded line above traces indicate p < 0.05. ZT: Zeitgeber time. VEH: vehicle injection. DZP: diazepam injection.

Next, we investigated the effects of diazepam injection on EEG spectra and slow wave activity. Fig. 3A shows a representative EEG power spectrogram for an individual animal during a baseline day followed by the diazepam injection day, with the corresponding hypnogram shown above to indicate the vigilance state of the animal. During the light period of the baseline day animals showed high spectral power in the slow wave frequency range indicative of NREM sleep, while the dark period was dominated by higher frequencies indicative of wakefulness. Consistent with previous studies, power in the slow frequency range was reduced after diazepam injection (Fig. 3B). Plotting power spectra of the transitions between NREM sleep and REM sleep (Fig. 3C) showed that diazepam injection reduced power in slow frequencies during NREM sleep and increased low theta frequency power during REM sleep. The effects on SWA and theta power were most prominent in the frontal and occipital EEG channels, respectively. Average power spectra for both the frontal and occipital cortical EEG were computed separately for wake, NREM sleep and REM sleep (Fig. 3B), during both injection days. In all cases repeated measures ANOVA’s (Greenhouse-Geisser) revealed a highly significant effect of frequency. Both EEG derivations also had a significant interaction between frequency and injection condition for both NREM sleep (frontal: F(1.8, 18.1) = 4.96, p = 0.02, p < 0.05 for frequency range 1–2.5 Hz and 4–4.25 Hz; occipital: F(1.6, 19.2) = 3.92, p = 0.05, no significant bins post hoc, Fig. 3B) and REM sleep (frontal: F(1.7, 17.2) = 4.98, p = 0.02, p < 0.05 for frequency ranges 5.25–6 Hz and 7.75–9.75 Hz; occipital: F(1.6, 19.0) = 3.68, p = 0.05, p < 0.05 for frequency ranges 5.25–5.75 Hz and 9.25 Hz). In order to account for variations in spectral power between animals, NREM sleep spectra for the injection days were normalised to the vehicle day for each individual animal before averaging across animals. Subdividing the light period into 3 h intervals confirmed that slow frequencies during NREM sleep were reduced across the entire 12 h light period after diazepam injection (Fig. 3D), while the increase in power within higher frequencies (>10 Hz) only occurred during the first three hours after diazepam injection (Fig. 3D). Repeated measures ANOVA (Greenhouse Geisser) revealed a significant effect of frequency (F(1.2,23.1) = 22.54, p < 0.0001) but no effect of time interval (F(3,20) = 0.09, p = 0.96) or the interaction (F(3.5,23.1) = 0.14, p = 0.95).

As NREM sleep SWA is strongly associated with sleep homeostatic processes, we next investigated the time course of SWA across the 12-hour light period. During the baseline day (Fig. 3F, left), and after vehicle injection (Fig. 3F, right), animals showed the typically observed high initial SWA levels at the beginning of the light period, which dissipated across time (Fig. 3E and F). In contrast, after diazepam injection SWA was consistently reduced compared to baseline and was significantly reduced compared to the vehicle injection day (repeated measures ANOVA (Greenhouse Geisser) factors; time: F(3.0,30.2) = 20.9, p < 0.0001; injection condition: F(1,10) = 210.26, p < 0.0001; interaction: F(3.0,30.2) = 3.71, p = 0.02, p < 0.0001 at all-time intervals).

**Fig. 4:**
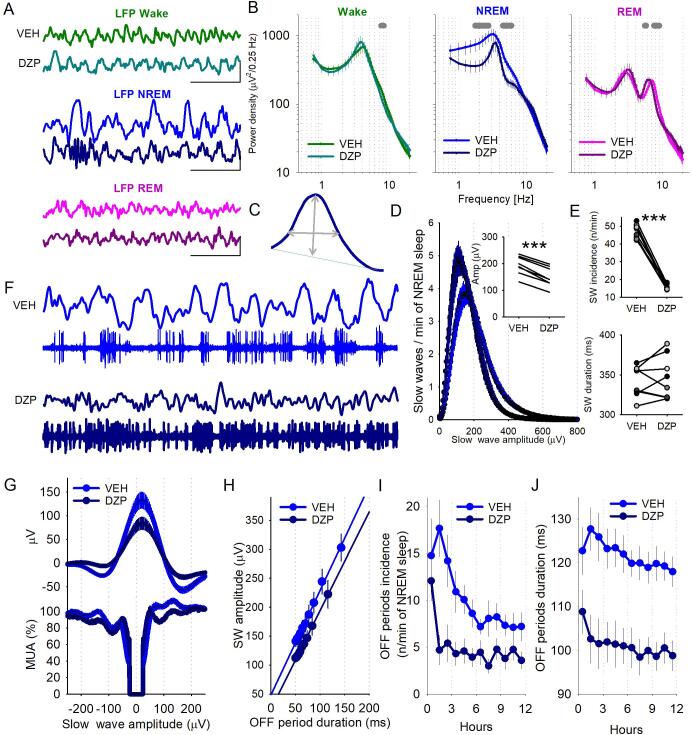
Effects of diazepam on LFP and OFF periods. A) Representative local field potential (LFP) signals during wakefulness (top), NREM sleep (middle) and REM sleep (bottom). For each vigilance state, the top and bottom traces represent signals from vehicle and diazepam injection days, respectively. Scale bars represent 1 s and 500 μV. B) Average LFP power spectra during wake (left, green), NREM sleep (middle, blue) and REM sleep (right, pink), are shown for vehicle and diazepam injection days. Repeated measures ANOVA with factors frequency, injection condition and their interaction (Wake: freq F(1.1,14.8) = 9.18, p = 0.008, injection condition F(1,14) = 2.97, p = 0.11, interaction F(1.1,14.8) = 5.82, p = 0.03; NREM: freq F(1.4,19.6) = 14.48, p < 0.0001, injection condition F(1,14) = 0.06, p = 0.82, interaction F(1.4,19.6) = 6.28, p = 0.01; REM: freq F(1.1,15.1) = 9.45, p = 0.007, injection condition F(1,14) = 2.11, p = 0.17, interaction F(1.1,15.1) = 5.69, p = 0.03). Shaded areas above traces indicate p < 0.05. n = 8. C) Schematic of an individual slow wave showing how amplitude and duration were quantified. D) The number of slow waves per minute are plotted as a function of their amplitude. n = 8. Note: diazepam injection was associated with a larger proportion of small amplitude slow waves as compared to vehicle injection. Inset: peak amplitude of slow waves during each injection condition. Paired *t*-test ***p < 0.0001. E) Average slow wave incidence (top) and duration (bottom). Paired t-tests. Amplitude ***p < 0.0001; duration p = 0.45. n = 8. F) Representative LFP and associated multiunit activity during NREM sleep after vehicle (top) and diazepam (bottom) injection. Note: periods of neuronal silence (OFF periods) are clearly identifiable after vehicle but not diazepam injection. G) Average LFP aligned to the midpoint of all OFF periods lasting at least 50 ms. MUA was normalised to the mean 300 ms before the OFF period. H) The relationship between the duration of OFF periods and slow wave amplitude. n = 6. I) J) Average OFF period incidence (I) and duration (J) per hour during the 12 h light period following injection. Repeated measures ANOVA; factors ‘time’, ‘Injection condition’ and their interaction. (incidence: time F(2.8,28.1) = 6.50, p = 0.002, injection condition F(1,10) = 10.12, p = 0.01, interaction F(2.8,28.1) = 2.30, p = 0.10; duration: time F(3.5,35.1) = 4.45,p = 0.007, injection condition F(1,10) = 12.86, p = 0.005, interaction F(3.5,35.1) = 1.54, p = 0.22). n = 6. In all panels dark blue and light blue represent the vehicle and diazepam injection days, respectively.

Next, we investigated the impact of diazepam on cortical neuronal activity and associated local field potentials recorded from deep layers of the motor cortex. This allows for the effects of diazepam on localised cortical regions to be disentangled from EEG recordings, which reflect the global behavioural state. Fig. 4A shows representative LFP traces during wake, NREM sleep and REM sleep, which can be defined using the classical criteria used for scoring the EEG into vigilance states (for example the presence of slow waves is indicative of NREM sleep). LFP power spectra replicated the effects of diazepam injection observed in the EEG (Fig. 4B). Most notably there was a consistent reduction in slow frequencies during NREM sleep (repeated measures ANOVA (Greenhouse-Geisser) factors ‘frequency’: F(1.4,19.6) = 14.48, p < 0.0001; ‘frequency*Injection condition’: F(1.4,19.6) = 6.28, p = 0.01, but no significant effect of ‘Injection condition’: F(1,14) = 0.06, p = 0.82, p < 0.05 for frequency ranges 1.75–2.75 Hz and 4.5–6 Hz). Both wake and REM sleep power spectra were also significantly affected by diazepam injection (wake: factors ‘frequency’: F(1.1,14.8) = 9.18, p = 0.008; ‘frequency*Injection condition: F(1.1,14.8) = 5.82, p = 0.03; p < 0.05 for frequency range 7.5–8.25 Hz; REM sleep: factors ‘frequency’: F(1.1,15.1) = 9.45, p = 0.007; ‘frequency*Injection condition: F(1.1,15.1) = 5.69, p = 0.03, p < 0.05 for frequency ranges 5.5–5.75 Hz and 7.5–9 Hz). Individual slow waves were identified and their incidence, amplitude and duration were quantified (schematic of an individual slow wave showing how amplitude and duration were quantified is shown in Fig. 4C). Diazepam injection resulted in a larger proportion of low amplitude slow waves, compared to vehicle injection (p < 0.0001, paired *t*-test, Fig. 4D). On average, across the 12 h light period slow waves were similar in duration but smaller in amplitude after diazepam injection (dur: p = 0.45; amp: p < 0.0001, paired *t*-test; Fig. 4E).

Slow waves are well established to be underpinned by regularly occurring periods of neuronal silence called OFF periods [10], [12], [52], [56]. After diazepam injection, this association was less distinct and the signals became asynchronous with one another as shown in the representative traces in Fig. 4F. To investigate this further we calculated the average LFP signal and aligned this to the midpoint of all OFF periods longer than 50 ms (Fig. 4G). As expected, we observed that OFF periods were associated with LFP slow waves; however, the amplitude of these were reduced after diazepam. Furthermore, while the duration of an OFF period was strongly positively correlated with the amplitude of slow waves, after both vehicle and diazepam injections (Fig. 4H), OFF periods were less frequent (repeated measures ANOVA (Greenhouse-Geisser): ‘time’: F(2.8,28.1) = 6.50, p = 0.002, ‘Injection condition’: F(1,10) = 10.12, p = 0.01, interaction: F(2.8,28.1) = 2.30, p = 0.10) and shorter in duration (repeated measures ANOVA: ‘time’: F(3.5,35.1) = 4.45,p = 0.007, ‘Injection condition’: F(1,10) = 12.86, p = 0.005, interaction: F(3.5,35.1) = 1.54, p = 0.22) after diazepam injection (Fig. 4I and J).

**Fig. 5:**
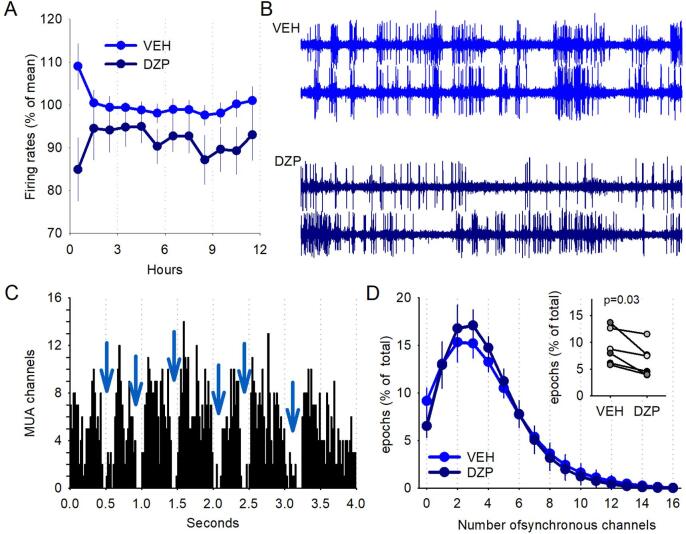
Effects of diazepam on cortical neuronal activity and spatial synchrony. A) Overall cortical firing rate during NREM sleep was calculated across all recording channels expressed a percentage of the mean during the vehicle injection light period. Repeated measures ANOVA (time F(2.2,21.8) = 0.42, p = 0.68, injection condition F(1,10) = 4.98, p = 0.05, interaction F(2.2,21.8) = 1.17, p = 0.33). n = 6. B) Multiunit activity traces from two recording channels from a representative animal during both the vehicle and diazepam injection day. Note: After diazepam injection multiunit activity becomes less synchronous across recording channels. C) The number of active recording channels with at least 1 spike plotted for consecutive 20 ms windows. Representative 4 s epoch in NREM sleep is shown in one individual animal. Note that during the majority of 20 ms bins at least some of the channels are active, but at times all channels are inactive simultaneously indicative of OFF periods (arrows). D) The distribution of the 20-ms epochs in NREM sleep plotted as a function of simultaneously active channels, expressed as percentage of total (light blue: vehicle, dark blue: diazepam, n = 6). Note that the number of 20-ms epochs with 0 active channels is significantly smaller after diazepam as compared to vehicle (shown as inset for individual animals). Wilcoxon sign rank test, p = 0.03.

As OFF periods are the result of a synchronous silence in neuronal activity across all recording channels, one possibility is that the neocortex is overall more active after diazepam treatment, or synchronicity between channels is reduced. We first calculated overall average firing rates across all recording channels expressed a percentage of the mean during the vehicle injection light period. Contrary to our expectation, we found that neuronal activity was somewhat lower during NREM sleep after diazepam, as compared to vehicle (repeated measures ANOVA (Greenhouse-Geisser): ‘Injection condition’: F(1,10) = 4.98, p = 0.05) although it was stable across the 12 h light period (time: F(2.2,21.8) = 0.42, p = 0.68; interaction: F(2.2,21.8) = 1.17, p = 0.33; Fig. 5A). Then, we addressed the second possibility of a reduced spatial synchrony between neurons recorded from the microwire array. Visual inspection of raw multiunit traces after diazepam revealed that it was not uncommon that neighbouring channels showed very different levels of activity at the same time, such that one channel may show intense neuronal activity while another just a few hundred microns away is in complete silence (Fig. 5B). We therefore quantified the number of recording channels (out of a total of 16 recording channels) that showed multiunit activity at the same time in 20 ms bins, to assess the degree of synchrony of neuronal activity across the network (Fig. 5C and D). The key observation was that on fewer occasions all recording channels were simultaneously silent after diazepam as compared to vehicle injection (p = 0.03; Wilcoxon sign rank test., Fig. 5D, inset). Apart from that, we found no significant differences between injection conditions for the number of channels contributing to the multiunit activity as a percentage of 4 s epochs (repeated measures ANOVA (Greenhouse-Geisser) on log transformed data: channels: F(1.9,18.7) = 59.62, p < 0.0001; Injection condition: F(1,10) = 0.74, p = 0.41; interaction: F(1.9,18.7) = 0.53, p = 0.59).

## 4 Discussion

The profound reduction in EEG spectral power in the SWA range with diazepam injection reported in this study is consistent with previous literature [29], [28], [34], [35], [79]. The influence of diazepam on EEG spectra was more apparent in the frontal region compared to the occipital, corresponding well with similar previous reports [28], [29]. The reduction in SWA with diazepam injection was maintained across the 12 h after diazepam. This is consistent with evidence in humans that showed the reduction in SWA persisted into the following drug-free night after injection of the benzodiazepines flunitrazepam, flurazepam or triazolam, whereas the effects on the spindle frequency range were recovered [8]. Diazepam also shifted the theta peak to slower frequencies, especially in the occipital derivation where theta activity is known to predominate due to its proximity to the hippocampus. It is possible that the shift in theta peak may reflect changes in body temperature, which have been previously associated with changes in EEG frequencies in animal and human studies [15], [16]. This is supported by evidence that diazepam decreases body temperature [38], and so hypothermia may have an influence on overall EEG frequencies and power spectra. However, the effects we observed in this study were state- and frequency-specific rather than resulting in a shift in overall power across all frequencies. Consistently, evidence in humans suggests that under certain conditions, physiological (e.g. circadian) changes in EEG power spectra may be dissociated from unspecific effects of body temperature [17]. We therefore believe that the effects we report here primarily reflect changes in neural activity induced by diazepam, rather than just being an indirect effect of hypothermia. Future studies should further investigate the association between diazepam induced hypothermia and EEG power spectra. We would like to add that when average NREM sleep spectra were subdivided into 3 h intervals, this revealed enhanced power in higher frequencies, including spindle-frequency range, during the first 3 h after diazepam injection only, while the effects on slow frequencies persisted at least until the end of the day, in line with previous studies [29], [28], [34], [35], [79].

While the molecular mechanisms of action for benzodiazepines have been elucidated in great detail, previous studies have not looked at the cortical neural activity underlying the response to diazepam in the neocortex. To our knowledge, this is the first study to investigate the neuronal activity underlying the effects of diazepam on localised cortical activity, as determined by local field potential and multiunit activity recordings from deeper layers of the primary motor cortex. Local field potential slow waves during NREM sleep were of smaller amplitude after diazepam injection, while the OFF periods associated with these slow waves were less frequent and shorter in duration. It is possible that this may be due to a reduced synchronicity across the cortex, which leads to fewer occasions where all channels are silent (OFF period) at the same time. This corresponds well with the known association between slow waves and their neuronal counterparts, where lower SWA spectral power is a result of the reduced occurrence of slow waves and population OFF periods [18], [54], [60], [75], [76], [84], [85]. Finally, although overall firing rates were lower after diazepam injection compared to vehicle injection, the more striking finding of this study was that on fewer occasions all recording channels were synchronously silent after diazepam injection. This indicates that diazepam may disrupt the synchronicity of neuronal activity across the cortex and therefore localised cortical activity may be differentially affected by diazepam, as compared to more global EEG mechanisms. This is supported by recent evidence that diazepam administration in mice induces a slowing of global electroencephalogram (EEG) oscillations in a frequency- and behavioural-state dependent manner [69].

It is now widely accepted that sleep is a cortical circuit phenomenon initiated locally, and gradually encompassing more networks across sleep periods [23], [32], [33], [36], [55], [67], [81]. Localised occurrence of sleep-like activity in distinct cortical regions during wakefulness (so called local sleep) has been shown both in rats and humans [11], [19], [46], and has been previously associated with deficits in cognitive performance in both animal and human studies [13], [26], [83]. Human intracortical recordings, performed simultaneously from multiple brain regions, have shown that slow waves during sleep are mostly confined to local regions, with typically ~ 30% of the brain showing simultaneous slow waves [47]. A number of species have adapted their sleep-wake cycle to express more localised forms of sleep to overcome evolutionary pressures such as migratory birds showing microsleeps [57], [58], or aquatic species sleeping one hemisphere at a time [43], [44], [59]. As adaptations such as these are fairly uncommon, it may be that the benefits associated with sleeping one hemisphere at a time are only relevant under specific extreme circumstances [58]. It is possible therefore, that local sleep does not provide the same benefits as regular, more global sleep [81]. Therefore, the effect of diazepam in disrupting the overall synchronisation of cortical activity may explain the observed effects of benzodiazepines on behaviour and cognitive function [27], [30], [66].

Despite these major effects of diazepam on EEG spectra and neuronal activity, this study and others [29] have reported little or no effect of diazepam on the total amount of NREM sleep in laboratory mice. This suggests that overall vigilance states may be differentially affected by diazepam, as compared to more localised cortical activity, and highlights the necessity to consider all levels of organisation when investigating the effects of sedative drugs on sleep quality. It remains to be established, whether the differences, or lack thereof, reported here may be generalised across other benzodiazepines and other cortical and subcortical brain areas, such as associative and sensory areas. As slow waves have been observed in every cortical region recorded to date, it is possible that the observations in the motor cortex could generalise to other cortical areas.

In conclusion, this study replicated the well-established effect of diazepam of reducing EEG SWA during NREM sleep and enhancing spectral EEG power in high frequency ranges. In addition, our findings suggest that these effects may be the result of a disruption in the synchronicity of neural activity across the cortex during NREM sleep which ultimately leads to a reduction in slow wave amplitude (and therefore reduced SWA) as a result of reduced incidence and duration of OFF periods associated with slow waves. This highlights the necessity to consider neural activity on multiple scales when investigating the effects of sedative and hypnotic drugs on sleep regulation and function. It may be that local and global mechanisms are differentially affected, which could have important consequences for brain recovery and memory processing during sleep and therefore should be considered in future studies.

## CRediT authorship contribution statement

**Laura E. McKillop:** Data curation, Formal analysis, Investigation, Methodology, Project administration, Software, Writing - original draft, Writing - review & editing. **Simon P. Fisher:** Investigation, Methodology. **Linus Milinski:** Investigation, Methodology, Writing - review & editing. **Lukas B. Krone:** Formal analysis, Methodology, Software, Writing - review & editing. **Vladyslav V. Vyazovskiy:** Conceptualization, Data curation, Formal analysis, Funding acquisition, Project administration, Resources, Software, Supervision, Writing - original draft, Writing - review & editing.

## Declaration of Competing Interest

The authors declare that they have no known competing financial interests or personal relationships that could have appeared to influence the work reported in this paper.
